# Sexual Signaling and Immune Function in the Black Field Cricket *Teleogryllus commodus*

**DOI:** 10.1371/journal.pone.0039631

**Published:** 2012-07-09

**Authors:** Jean M. Drayton, Matthew D. Hall, John Hunt, Michael D. Jennions

**Affiliations:** 1 Evolution, Ecology and Genetics, Research School of Biology, The Australian National University, Canberra, ACT, Australia; 2 Evolutionary Biology, Zoologisches Institut, University of Basel, Basel, Switzerland; 3 College of Life and Environmental Sciences, Centre for Ecology and Conservation, University of Exeter in Cornwall, Penryn, United Kingdom; University of Plymouth, United Kingdom

## Abstract

The immunocompetence handicap hypothesis predicts that male sexual trait expression should be positively correlated with immunocompetence. Here we investigate if immune function in the cricket, *Teleogryllus commodus,* is related to specific individual components of male sexual signals, as well as to certain multivariate combinations of these components that females most strongly prefer. Male *T. commodus* produce both advertisement and courtship calls prior to mating. We measured fine-scale structural parameters of both call types and also recorded nightly advertisement calling effort. We then measured two standard indices of immune function: lysozyme-like activity of the haemolymph and haemocyte counts. We found a weak, positive relationship between advertisement calling effort and lysozyme-like activity. There was, however, little evidence that individual structural call components or the net multivariate attractiveness of either call type signalled immune function. The relationships between immunity and sexual signaling did not differ between inbred and outbred males. Our data suggest that it is unlikely that females assess overall male immune function using male calls.

## Introduction

Female mate choice based on male sexual ornaments and displays is well documented, but the function of most mating preferences is poorly understood. Choosiness is selected for if preferred sexual traits signal aspects of male ‘quality’ that benefit choosy females (review: [1]). Direct benefits accrue to a choosy female if non-random mating increases her longevity or fecundity. In addition, indirect benefits arise if there are paternal effects on a choosy female’s progeny due to inheritance of genes [2] and/or paternal contributions (e.g. increased male care; [3]) that elevate offspring fitness. The mechanisms that allow sexual traits to function as reliable signals of male quality generally require that they are costly to produce or maintain (review: [4]), and that higher quality males pay a smaller marginal cost to increase investment ([5], [6] but see [7], [8]).

A specific case study of the costs of sexual signaling is related to a potential trade-off between sexual trait expression and immune system function. The original hypothesis was formulated for vertebrates under the assumption that testosterone, which is needed to produce male secondary sexual traits, is an immunosuppressant ([9] but see [10]), but other mechanisms could also generate the required trade-off [11]. In insects an immunosuppressive cost of sexual signaling could reflect a resource trade off: sexual displays require resources (e.g. energy, nutrition) that are diverted away from the immune system [12]. Alternatively, immune system activation could reduce investment into sexual signals [13]. Only males with sufficient resources (i.e. in good condition) can invest in costly sexual display and maintain a strong immune system. If so, the expression of male sexual signals will, given certain assumptions (see [14]), be positively correlated with immunocompetence. A trait that signals immunocompetence is a potentially important reason for females to engage in mate choice. Mating with males with greater resistance could have direct benefits (e.g. lower risk of infection while mating) or indirect benefits if disease resistance is heritable. Indirect benefits will be gained if the offspring of more attractive males are of above average immunocompetence (e.g. [15] but see [16]), and immunocompetence is positively correlated with net fitness [1]. There is some evidence for additive genetic variation in measures of immune function in insects (e.g. [15]–[20]). This could be due to genes that directly enhance immune function, or arise indirectly via genes that have a positive effect on body condition [11].

Invertebrates are popular subjects for studies of parasite-mediated sexual selection. Studies of invertebrates have reported phenotypic correlations between male sexual signals and measures of immune function that are both positive (beetles: [21], [22], crickets: [23]–[25], damselflies: [26]–[29], spiders: [30], scorpionflies: [31]) and negative/absent [23], [24], [26], [27], [29]–[34]. Given these contrasting results, more data is needed to quantify general relationships and identify sources of variation.

Here we test whether sexual traits signal male immune function in the cricket, *Teleogryllus commodus*. Males produce an advertisement and then a courtship call prior to mating. The advertisement call attracts sexually receptive females toward the male. Once a female has approached, the male produces a courtship call to induce mating. Female choice exerts strong positive directional sexual selection for greater advertisement calling effort and moderate levels of stabilizing and directional multivariate sexual selection on finer-scale advertisement call structure [35], [36]. Furthermore, dietary manipulation has shown that advertisement calling effort is affected by nutrition [37], [38]. Importantly, inbreeding reduces advertisement calling effort [39] and alters fine scale advertisement call structure [40], indicating that both call effort and call structure might signal male fitness (given that inbreeding lowers fitness). It is therefore possible that advertisement calls signal key fitness components such as disease resistance.

The fine-scale structure of the courtship call is under post-copulatory sexual selection because its affects spermatophore retention [41], which determines the number of sperm transferred [42], [43]. Inbreeding also affects courtship call structure [44], indicating that courtship calls might signal fitness to females. There is evidence from the congeneric *T. oceanicus* that courtship calls signal aspects of immune function [45], although this does not appear to be the case in another cricket, *Gryllodes sigillatus* [33]. Crucially, however, these studies only examined the relationship between individual components of the courtship call and immune function. This is biologically problematic as selection rarely acts on single call traits in isolation (review: [46]). To determine if male calls signal immune function to females during mate choice it is prudent to quantify multivariate combinations of call traits that are the direct target of mate choice.

We investigated whether advertisement or courtship calling signals male immune function in *T. commodus*. We recorded two measures of immune function: lysozyme-like activity in the haemolymph and haemocyte counts. Lysozyme is an antibacterial enzyme that hydrolyses bacterial cell walls. Its activity is assayed by measuring the rate at which an individual’s haemolymph clears a bacterial suspension (e.g. [47]). Haemocytes are cells in the haemolymph, and a subset of these are involved in immune responses by transporting molecules to infection sites, by ingesting foreign particles, or by encapsulating parasites [14], [48]. We first tested for relationships between 11 fine-scale characteristics of advertisement or courtship calls and male immune function. The five advertisement call traits that we measured were previously used in a selection analysis of *T. commodus* to quantify multivariate sexual selection due to female choice [35]. Similarly, the six courtship call traits were previously used to quantify multivariate sexual selection arising from cryptic female choice for spermatophore retention [41].

We utilized these selection analyses in conjunction with the measured call traits to estimate the multivariate attractiveness of each male’s calls. We then tested whether attractiveness signals male immune function. We also tested for any relationship between the time spent advertisement calling each night (hereafter ‘calling effort’) and immunity. Males who call more are more attractive to females [36]. Finally, we have already shown that inbreeding between full siblings affects both immune function [49] and male calling [39], [40], [44]. Inbreeding might therefore decrease the reliability of male calling as a signal of immune function by, for example, disrupting the usual proximate mechanism that determines the appropriate allocation of resources to calling and immune function. Consequently, we tested whether the relationship between calling and immunity differed between inbred (*F* = 0.25) and outbred males. In sum, we asked three key questions:

1. Do fine-scale structural features of courtship or advertisement calls signal male immune function (lysozyme activity or haemocyte count)?
2. Do the combinations of male call characteristics that most strongly influence female choice (i.e. the multivariate attractiveness of each call type) or total calling effort signal male immune function?
3. Does inbreeding weaken the relationship between call traits and immunity? If not, the previous two questions can be addressed with a pooled dataset.

## Methods

### General Breeding Protocols

We collected ∼70 wild caught gravid females from Smiths Lake, New South Wales, Australia in May 2006. No specific permits were required for the collection of the crickets from the field site. The site where the crickets were collected is not privately-owned or protected in any way. *T. commodus* is not an endangered or protected species. The offspring of the wild caught females (i.e. half-siblings) were reared to adulthood. Each family was maintained separately. From these half-sibling families, we randomly paired virgin males and females (pair members did not have the same wild caught mother). Each of the resultant full-sibling families were reared in separate 43×30×13 cm plastic tubs with dry cat food (KiteKat Krunch, Uncle Ben’s, Raglan, Australia) and water provided *ad libitum*. Crickets were maintained at 26–28°C on a 12∶12 photoperiod.

**Figure 1:**
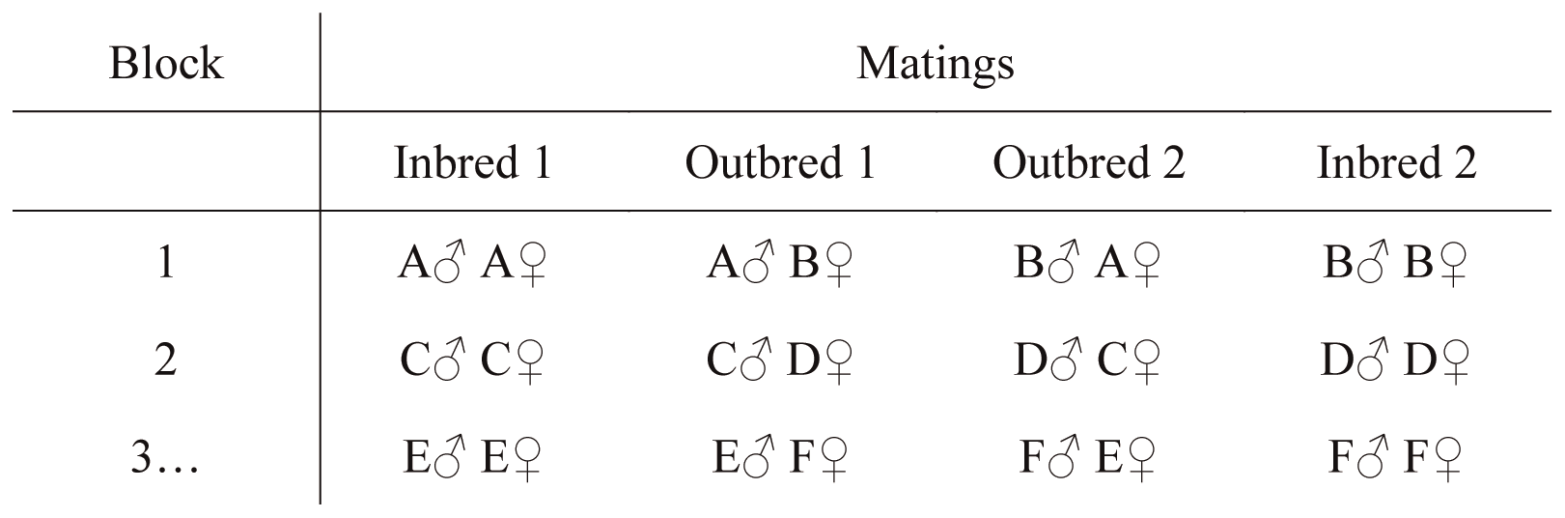
The mating design. Block 1 is comprised of full-sib families A and B, Block 2 is comprised of full-sib families C and D, and so on. There are two inbred and two outbred genotypes generated per block.

The full-sibling families were grouped into pairs, hereafter known as blocks (*N* = 33 blocks, Fig. 1). The two families in each block were always unrelated. Each full-sibling family was only used once (i.e. 66 full-sibling families in the 33 blocks). In each block, brothers and sisters from both full-sibling families were mated to create two inbred genotypes (*F* = 0.25). Outbred genotypes (*F*≈0) were created by reciprocal matings between families in the block. For example, in a block consisting of full-sibling families A and B, inbred progeny were generated by mating brothers and sisters from family A, and brothers and sisters from family B, while outbred progeny were generated by mating an A family male with a B family female, and a B family male with an A family female (Fig. 1). This design generated four offspring genotypes per block (two inbred; two outbred).

After mating females were provided with moist cotton wool (“egg pads”) for egg laying. Egg pads were checked every three days for emerging nymphs. Upon emerging, nymphs from the same pairing were reared communally for 20 days in 9×9×5 cm plastic tubs with food and some moist cotton wool. After 20 days, each nymph was transferred to an individual 9×9×5 cm container with a small tube of water plugged with cotton wool, a piece of cat food and a cardboard egg cup. Nymphs were not transferred immediately after emerging because they were small enough to be crushed by the water tube. The density of nymphs during the communal stage was similar in inbred and outbred groups. It is unlikely that communally reared nymphs competed for resources because they were very small, so densities were low, and food and water was provided *ad libitum*. Once crickets were set up individually, food and water was replaced every 10 days. On average, 37.5±3.3 (± SE) nymphs of each inbred genotype and 39.4±3.3 nymphs of each outbred genotype were set up individually for each block. All the measured males were >10 days post maturation to ensure that they were sexually mature.

### Measuring Immune Function

We used a sterile pin to make a small puncture under the pronotum (posterior, dorsal edge) to collect 2 µl of haemolymph with a Gilson pipette, which was added to 8 µl of phosphate-buffered saline (PBS: 8 g NaCl, 0.2 g KCl, 1.44 g Na_2_HPO_4_, 0.24 g KH_2_PO_4_ in 1 L distilled water, pH 7.4), and frozen to induce cell lysis. The samples were later thawed and 90 µl of a *Micrococcus lysodeikticus* bacterial solution (3 mg/ml PBS) added. We then loaded samples into a microplate spectrophotometer (PowerWave 340, Bio-Tek Instruments Inc.) to measure lysozyme activity at 490 nm and 30°C as the rate of change in optical density from 0 to 80 minutes. Several control samples (a PBS and *M. lysodeikticus* solution) were loaded on each plate to control for variation among assays. The rate of change in optical density for each haemolymph sample was calculated as the sample slope minus the control slope. This was multiplied by −1 so that a greater value indicates higher lysozyme-like activity. We also collected another 2 µl of haemolymph from the pronotum puncture and mixed it with 8 µl of anticoagulant (3.92 g NaOH, 8.532 g NaCl, 6.328 g EDTA, 8.616 g citric acid in 1 L distilled water). This mixture was expelled onto a Neubauer haemocytometer and we estimated the number of haemocytes per ml of haemolymph. Lysozyme activity and haemocyte counts are repeatable (lysozyme: *r*_I_ = 0.74, *n* = 161; haemocytes: *r*_I_ = 0.52, *n* = 56, both *P*<0.001), and are positively correlated across individuals (*r* = 0.56, *P*<0.001, *n* = 675) [49]. Males were weighed (±0.1 mg) before haemolymph extraction and were 23.0±0.10 days post-maturation.

### The Advertisement Call

**Figure 2:**
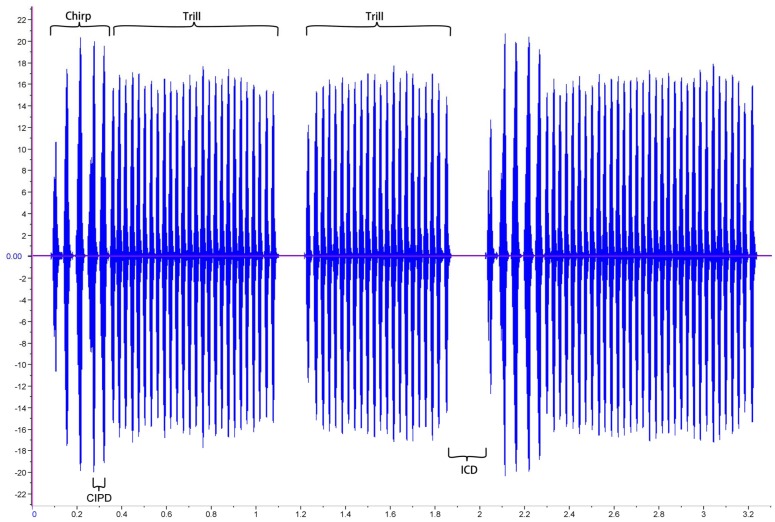
The advertisement call of *T. commodus*. Each phrase consists of a single chirp followed by a variable number of trills (the phrase in the figure has two trills). We measured: dominant frequency (DF), chirp pulse number (CPN), chirp inter-pulse duration (CIPD), inter-call duration (ICD), and trill number (TN). The vertical axis represents the amplitude of the sound produced.

Males call using a stridulatory apparatus consisting of a file and scraper on the forewings. The most basic call unit is a pulse, produced by closing the wings once [50]. There are two types of sound pulses in *T. commodus* advertisement calls. The first are longer, more intense and grouped into chirps. The second are shorter, softer and grouped into trills. Chirps and trills are arranged into the main repeated unit of the call, a phrase, which is a chirp followed by one to several trills [51], [52] (Fig. 2).

Males were set up in individual, acoustically isolated 9×9×5 cm recording chambers with a condenser microphone mounted in the lid that was powered by a 9 volt battery. We recorded calls with a digital recorder (MicroTrack 24/96, M-Audio, Irwindale, CA, USA). Chambers were checked in the evenings for calling males. If a male was heard calling, we recorded him for 1–2 minutes, and then weighed him. Recorded males were 23.0±0.5 days post maturation.

Calls were analyzed using Raven Pro 1.3 (Cornell Lab of Ornithology, www.birds.cornell.edu/raven). We measured five parameters of five randomly chosen phrases from each male: dominant frequency (DF), the interval between the last trill pulse of the selected phrase and the first chirp pulse of the next [inter-call duration (ICD)], the number of pulses per chirp [chirp pulse number (CPN)], the duration of the interval between the last two pulses in the chirp [chirp inter-pulse duration (CIPD)] and the number of trills in a phrase [trill number (TN)] (Fig. 2). These were the same five call parameters used by Brooks et al. [35] to quantify multivariate sexual selection on advertisement call structure (see below). Calls were filtered before analysis to remove ambient noise below 3 kHz and above 6 kHz. Call parameters measured in this way are highly repeatable [39]. We therefore calculated a mean value of DF, ICD, CPN, CIPD and TN for each male. We measured advertisement call structure and lysozyme-like activity for 50 males, and call structure and haemocyte counts for 55 males.

### The Courtship Call

**Figure 3:**
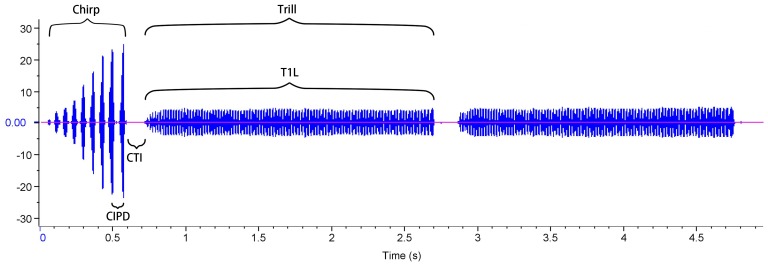
The courtship call of *T. commodus*. Each phrase consists of a single, amplitude-modulated chirp, followed by a variable number of trills (the phrase in the figure has two trills). We measured: dominant frequency (DF), chirp pulse number (CPN), chirp inter-pulse duration (CIPD), chirp-trill interval (CTI), trill number (TN) and the length of the first trill (T1L). The vertical axis represents the amplitude of the sound produced.

The courtship call also consists of pulses that are grouped into chirps and trills that are then arranged into repeated phrases. Each phrase consists of a single, amplitude-modulated chirp, followed by several trills that are consistent in intensity [41] (Fig. 3). Males were set up in individual 9×9×5 cm recording chambers with a condenser microphone mounted in the lid. To induce a male to call, a randomly chosen virgin stock female was introduced into the chamber. We recorded 1–2 minutes of courtship calling using the MicroTrack recorder (see above). The male and female were then weighed. Males were 20.8±0.2 days post maturation. Using the same protocol described for advertisement calls we measured six call parameters from five randomly chosen phrases per male: dominant frequency (DF), chirp pulse number (CPN), the chirp inter-pulse duration (CIPD), the duration of the interval between the chirp and the first trill [chirp-trill interval (CTI)], trill number (TN) and the length of the first trill [trill 1 length (T1L)] (Fig. 3). These were the same six call parameters used by Hall et al. [41] to estimate multivariate sexual selection on the structure of the courtship call (see below). Again, these call parameters are highly repeatable [44], so we calculated a mean value for each parameter per male. We measured courtship call structure and lysozyme-like activity for 204 males, and courtship call structure and haemocyte counts for 197 males.

### Calling Effort

Male nightly calling effort was measured using a custom-built electronic monitoring device (see [39]). In brief, the device consists of 128 recording chambers, grouped into sets of 16 chambers. Each chamber (9×9×5 cm) had a condenser microphone mounted in the lid and housed one male. The device sampled the chambers in sets of 16 throughout the night. During sampling, the microphone of one chamber in each set was activated. If the sound level was >10 dB above the background noise a 1 representing that the male was calling was recorded, otherwise, a 0 was recorded. The microphone was then de-activated and the microphone in the next chamber of a set was activated. Each recording chamber in a set was sampled 10 times per second. If a male was recorded as calling for any of the 10 sampling events/second, he was recorded as calling for that second. Nightly calling effort was defined as the total number of seconds called per night. Calling effort was measured for 10 hours/night (19h00–05h00). Males were weighed before they were put into a chamber. Males were 14.4±0.02 days post maturation, and calling effort was recorded for 3.2±0.01 nights. Calling effort is repeatable [39]. We therefore calculated mean nightly calling effort for each male. We measured calling effort and lysozyme activity for 511 males, and calling effort and haemocyte counts for 408 males. If a male registered a very low or even ‘0’ nightly calling effort, he was still included in the analyses.

In total, 622 males were used in the study. For each male, we tried to record as much information as possible about his level of immune function (lysozyme-like activity and haemocyte counts) and sexual signaling (i.e. record his advertisement and courtship call, and measure nightly calling effort). There is therefore overlap in the males used to test for different relationships. Due to logistical constraints, however, it was not possible to measure every parameter on every male. Consequently, the sample sizes for different tests vary, and are a reflection on the ease of measuring each parameter. For example, measuring calling effort was mostly an automated process that was done overnight using a custom built device. Therefore the sample sizes for calling effort are large (>400). Similarly, the sample sizes for courtship calls are high as these calls were easily induced in males by introducing a female to the recording chamber (>200). In contrast, advertisement calls were recorded opportunistically, and males can not be made to produce an advertisement call on demand. So, despite intensive efforts, our sample sizes for advertisement call structure are modest. For all males, we first recorded sexual signaling (advertisement call, courtship call and/or calling effort), and then immune function was assayed afterwards.

### Estimating Multivariate Call Attractiveness

Both the courtship [41] and advertisement [35] call of *T. commodus* are subject to strong sexual selection due to female choice. Hall et al. [41] showed that the time until an unguarded female removes a male’s spermatophore (thus terminating sperm transfer) can be predicted based on the structure of his courtship call. The strongest vector of sexual selection on male courtship call structure, **m_7_**, is characterized by stabilizing selection (Fig. 3 in [41]) and is described by the equation:
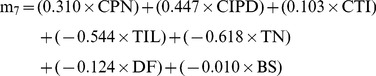
(1)


Thus by substituting our courtship call parameters and body size (BS) into this equation, we can derive an **m_7_** score for each male in our study. Furthermore, as sexual selection along this vector is stabilizing, we can solve for *Y* = *a*x^2^+*b*x+*c*, where *a* = −0.860 (**λ** in Table 3 of [41]), *b* = 0.027 (***θ***, linear selection along this vector) and *c* = 1.122 (the intercept from the quadratic regression of **m_7_** on relative fitness). For courtship calls, *Y* is our best estimate of a male’s multivariate post-copulatory attractiveness.

Likewise, Brooks et al. [35] showed in laboratory-based mate choice trials that the strongest vector of sexual selection on the advertisement call, **m_5_**, is subject to both linear and stabilizing selection, as convex selection is asymmetrical over the range of call structure components tested (Fig. 2 in [35]). This relationship is described by the equation:
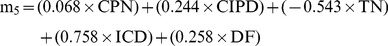
(2)


Again, we can substitute our advertisement call parameters into this equation to obtain an **m_5_** score for each male and solve for *Y* = *a*x^2^+*b*x+*c* to estimate each male’s multivariate pre-copulatory attractiveness. Here, *a* = −0.119, *b* = −0.160 (**λ** and ***θ*** respectively in Table 3 of [35]) and *c* = 1.038. Our approach to construct a multivariate measure of attractiveness is similar to that used by Jang and Greenfield [53].

### Statistics

To characterize the relationship between immunity and male sexual signals we ran multiple regressions with call properties or male attractiveness as continuous predictor variables and inbreeding treatment as a categorical variable. Before analysis we transformed average calling effort, trill number (TN) and inter-call duration (ICD) using natural logarithms, and lysozyme activity, haemocyte counts, chirp to trill interval (CTI) and first trill length (T1L) using square root transformations. Transformations were performed to ensure multivariate normality. In addition, we standardized (mean = 0, SD = 1) the structural parameters of the courtship and advertisement call to maintain consistency with the previous selection analyses [35], [41] and enable us to estimate the multivariate attractiveness of calls (i.e. to correctly apply equations 1 and 2 above). We scaled our immune measures to relative estimates by dividing by the transformed mean, and standardized all other traits (mean = 0, SD = 1). The use of relative immune estimates and standardized predictor traits makes it easier to directly interpret differences between regression coefficients, as traits were measured on different scales.

We then used partial *F*-tests to compare the fit of more complex regression models to reduced alternatives [54]. We evaluated the potential for linear relationships between immune measures and the predictor traits of interest (linear compared to intercept only model) and, based on the addition of linear by inbreeding treatment interaction terms, we tested if the relationships differed between inbred and outbred males (interaction compared to linear only model). All analyses were originally performed with block as a random factor. However, block had no effect on the regression coefficients, so we report the simplified analyses that excludes this term.

Given that inbreeding did not affect the relationship between male calling and immunity (see Results), we did not conduct separate analyses for inbred and outbred males to answer questions 1 and 2. The inbreeding term was, however, always retained in the final models because we have previously shown that inbreeding can affect immune parameters [49].

All analyses were conducted in JMP (Version 8.2, SAS, NC, USA). All test are two-tailed at α = 0.05. To aid comparison of the biological importance of different relationships, we calculated standardized effect sizes by converting the *F*-statistic into a common effect size (Pearson’s r) using the Metawin 2.0 calculator [55].

Finally, although not the main focus of our study, we tested for bivariate correlations (Pearson’s r) between the multivariate attractiveness of both the courtship and advertisement call, and calling effort. This allows us to test if different sexual signals provide separate information about male quality to females.

## Results

### 1. Does Fine-Scale Call Structure Signal Male Immune Function?

There was little evidence for a relationship between specific call structure characteristics and immune function. Individual call properties of both the advertisement call (Table 1) and courtship call (Table 2) were only weakly associated with both lysozyme activity and haemocyte count. We did not find any evidence for the overall ability of the five advertisement call characteristics to predict lysozyme activity (*F*_5, 43_ = 0.544, *P* = 0.742) or haemocyte count (*F*_5, 48_ = 1.323, *P* = 0.270), nor for the six characteristics of the courtship call to predict lysozyme activity (*F*_6, 196_ = 1.085, *P* = 0.372) or haemocyte counts (*F*_6, 189_ = 2.059, *P* = 0.060).

**Table 1:** The standardized linear regression coefficients describing the relationships between the properties of the advertisement call and the two different immune assays.

	Lysozyme activity		Haemocyte count	
Advertisement call	*β*	95% CI	*β*	95% CI
CPN	−0.068	(−0.217, 0.079)	−0.067	(−0.176, 0.040)
CIPD	0.029	(−0.111, 0.169)	0.034	(−0.073, 0.141)
TN	0.011	(−0.108, 0.130)	0.016	(−0.085, 0.117)
ICD	−0.053	(−0.189, 0.083)	−0.061	(−0.170, 0.047)
DF	0.076	(−0.062, 0.214)	0.110	(<0.001, 0.220)

CPN: chirp pulse number, CIPD: chirp inter-pulse duration, TN: trill number, ICD: inter-call duration, DF: dominant frequency.

**Table 2:** The standardized linear regression coefficients describing the relationships between properties of the courtship call and the two different immune assays.

	Lysozyme activity		Haemocyte count	
Courtship call	*β*	95% CI	*β*	95% CI
CPN	0.039	(−0.026, 0.104)	0.076	(0.011, 0.140)
CIPD	0.022	(−0.043, 0.086)	−0.032	(−0.097, 0.033)
CTI	−0.033	(−0.099, 0.034)	−0.028	(−0.094, 0.039)
T1L	−0.089	(−0.186, 0.007)	−0.022	(−0.119, 0.076)
TN	−0.095	(−0.187, −0.003)	−0.062	(−0.154, 0.030)
DF	0.018	(−0.045, 0.081)	0.033	(−0.029, 0.095)

CPN: chirp pulse number, CIPD: chirp inter-pulse duration, CTI: chirp-trill interval, T1L: the length of the first trill, TN: trill number, DF: dominant frequency.

### 2. Does Call Multivariate Attractiveness or Calling Effort Signal Immune Function?

The multivariate attractiveness of the advertisement call predicted neither lysozyme activity (*F*_1, 48_ = 0.021, *P* = 0.887) nor haemocyte count (*F*_1, 53_ = 0.132, *P* = 0.718). The same was true for the courtship call (lysozyme: *F*_1, 202_ = 1.394, *P* = 0.239; haemocyte count: *F*_1, 195_ = 1.848, *P* = 0.176). Calling effort was not significantly related to haemocyte count (*F*_1, 405_ = 1.432, *P* = 0.232). There was, however, a significant positive relationship between calling effort and lysozyme activity (*F*_1, 508_ = 5.957, *P* = 0.015) (Table 3).

**Table 3:** The linear regression coefficients describing the relationships between the two different immune assays and three traits that have been shown to affect male mating/fertilization success.

	Lysozyme activity					Haemocyte count				
Attractiveness measures	*β*	*SE*	P	r (95% CI)	n	*β*	*SE*	P	r (95% CI)	n
Courtship call attractiveness	0.019	0.016	0.239	0.082 (−0.057 to 0.219)	204	0.022	0.016	0.176	0.096 (−0.046 to 0.235)	197
Advertisement call attractiveness	−0.072	0.504	0.887	0.020 (−0.257 to 0.944)	50	0.153	0.422	0.718	0.049 (−0.218 to 0.308)	55
Calling effort	0.046	0.019	0.015	0.108 (0.020 to 0.194)	511	0.026	0.022	0.232	0.059 (−0.040 to 0.157)	408

The effect size (*r*) is shown. For a given immune assay, each coefficient was estimated using separate regressions, but represented here within a single table for simplicity. P-values significant at the 0.05 level are in bold.

As the sample size used to examine the relationship between calling effort and lysozyme activity was considerably larger than those involving call structure, we tested the robustness of this analysis using a bootstrapping procedure where we limited the number of males involved in the regression to the minimum number used in the other analyses (i.e. 50 individuals) and then resampled the data 10,000 times without replacement to estimate the 95% confidence intervals. The 95% confidence intervals for the regression gradient ranged from −0.068 (lower 95% CI) to 0.160 (upper 95% CI) and thus overlapped zero. This suggests that the relationship between lysozyme activity and calling effort is actually quite weak and the statistical significance we detected is contingent on the large sample size used in our study.

### 3. Does Inbreeding Weaken the Relationship between Calling and Immunity?

Male inbreeding status did not change the relationships between either call type and immune function. For the advertisement call, there was no difference between inbred and outbred males in how the five call characteristics were related to each immune assay (lysozyme: *F*_5, 38_ = 0.319, *P* = 0.899; haemocyte: *F*_5, 43_ = 0.581, *P* = 0.714). Likewise, for the courtship call, there was no difference between inbred and outbred males in how the six call characteristics were related to each immune assay (lysozyme: *F*_6, 190_ = 0.901, *P* = 0.495; haemocyte: *F*_6, 183_ = 0.418, *P* = 0.866). Similarly, inbreeding did not change the relationships between the multivariate attractiveness of advertisement or courtship calls and either immune measure (advertisement: lysozyme: *F*_1, 46_ = 0.495, *P* = 0.485; haemocyte: *F*_1, 51_ = 0.507, *P* = 0.480; courtship: lysozyme: *F*_1, 200_ = 0.453, *P* = 0.502; haemocyte: *F*_1, 193_ = 0.142, *P* = 0.707). Finally, there was no effect of inbreeding on the relationship between male nightly calling effort and lysozyme activity (*F*_1, 507_ = 0.071, *P* = 0.791) or haemocyte count (*F*_1, 404_ = 2.249, *P* = 0.116).

### Correlations between Measures of Multivariate Attractiveness, and Calling Effort

There was no correlation between the multivariate attractiveness of the courtship call and the advertisement call (*r* = 0.146, *P* = 0.459, *N* = 28). Calling effort was not related to the multivariate attractiveness of either call (advertisement: *r* = 0.112, *P* = 0.270, *N* = 98; courtship: *r* = 0.078, *P* = 0.110, *N* = 417).

## Discussion

### Calling Effort

The immunocompetence handicap hypothesis [9] implies that the expression of male sexual traits will be positively correlated with immunocompetence. In *T. commodus* calling effort is moderately, positively related to lysozyme-like activity in the haemolymph. There was, however, no such relationship with haemocyte count, even though haemocyte count is strongly correlated with lysozyme activity (*r* = 0.56 [49]). A potential proximate mechanism generating a positive correlation between calling effort and lysozyme activity is that both traits are condition-dependent. Calling is energetically costly for crickets [50], [56], and in *T. commodus* advertisement calling effort is dependent on nutrition [37], [38]. Calling effort is also negatively affected by inbreeding, which tends to reduce body condition [39]. It is currently unknown whether lysozyme activity is affected by resource assimilation in *T. commodus*. Several studies have, however, shown that maintaining and upregulating the immune system is nutritionally and energetically demanding for insects (e.g. [57]–[61]). If the expression of lysozyme activity is condition-dependent, there is a trade off between resources allocated to lysozyme activity and calling effort [12], and consequently a negative relationship might be expected. Variation among males in resource acquisition and assimilation ability (i.e. condition) is, however, typically high ([62] see also [63]) and this often results in positive phenotypic relationships between condition-dependent traits at the population level (Box 2 in [64]) because, for example, only individuals in good condition, can invest in high levels of lysozyme production and maintain a high calling effort. Future studies that manipulate male condition using experimental diets (see [38], [65]) and then measure immune function are therefore a high priority.

Field studies have demonstrated that male *T. commodus* with a greater nightly calling effort attract more females [36]. Females might show an active preference for males that call more because calling effort is condition dependent [37] and could therefore signal genetic quality or direct benefits. It also might simply be easier for females to locate males that call more [36]. Regardless, a preference for males who call more increases the likelihood of obtaining a mate with a greater lysozyme activity, which could confer direct or indirect benefits to females. It is worth noting however, that the effect size for the relationship between calling effort and lysozyme activity is small (although not that different from many effect sizes reported for correlations of sexual traits with life history characters; Jennions et al., unpublished data). Furthermore, when we bootstrapped the data examining the relationship between calling effort and lysozyme-like activity using a sample size comparable to our analyses using call structure data (*n* = 50), we found that the 95% confidence intervals overlapped zero indicating that the statistical significance of this relationship is contingent on the large sample size we used. Therefore this relationship must be viewed with caution and a preference for increased calling effort may only slightly increase a female’s chance of mating with a male with above average lysozyme activity.

### Call Structure

We found little evidence that fine scale structural components of male courtship or advertisement calls were related to immune function either when considered individually, or as multivariate trait combinations that influence net male attractiveness. In contrast, other studies with crickets have reported that the structure and/or attractiveness of advertisement calls (e.g. [23], [25] but see [16]) and courtship calls (e.g. [24], [45] but see [33]) signal aspects of immunity. In particular, Simmons et al. [25] found that fine scale advertisement call structure provides some information about male immune function in another population of *T. commodus*. There were, however, several differences between our study and Simmons et al. [25] that do not make them directly comparable. First, they recorded advertisement calls from males from a field population, who were exposed to natural parasites and pathogens that could induce differences in immune function due to variation in parasite loads or past exposure to disease. We used laboratory reared males not exposed to parasites and measured innate immune function. Second, benign laboratory conditions, where there is *ad libitum* food and water, might mask correlations between call structure and immune function seen in the field that are mediated through variation in body condition associated with differences in resource acquisition [11]. Third, Simmons et al. [25] measured a different aspect of male immune function (encapsulation rate).

Finally, although the call traits that we measured influence female choice in our study population [35], [36], [41], we used the results of previous selection analyses that were conducted a few years earlier to predict the multivariate attractiveness of calls. We are therefore assuming that fitness surfaces are stable across time.

### Immune Measures

Haemocyte counts and lysozyme-like activity have both been used as a proxy for immunocompetence in invertebrates (e.g. [15], [16], [25], [30], [45], [66], [67]). It is important to note, however, that larger measured immune responses do not always indicate greater resistance against actual disease [68], [69]. In most studies of parasite-mediated sexual selection it is unknown whether the reported immune measure accurately reflects disease resistance. The same is true in *T. commodus* for haemocyte counts and lysozyme activity. We have previously shown that inbreeding (which lowers fitness) actually increases one of our immune measures (haemocyte counts) demonstrating that the correlation between fitness and immune function is not necessarily linear and might involve an optimal level of defense [49]. It appears, however, that the elevated lysozyme activity of males who call more is associated with higher fitness as males with greater calling effort attract more females [36], which is likely to be a major component of net male fitness. Given our previous findings that inbreeding affects immune function [49] and calling effort [39], we tested whether the relationship between sexual signals and immune function differed between inbred and outbred males. This might occur, for example, if inbreeding affects the allocation of resources (e.g. energy, nutrients) between sexual signaling and the immune system. However, inbreeding did not affect any of the relationships between calling and the two immune measures. One interpretation is that inbreeding affects general body condition but not resource allocation. Given condition-dependence, this would explain how inbreeding can affect the absolute level of immune function [49] and calling effort [39], but not alter the relationship between the two traits (this study).

It is worth noting that there are two general approaches to measuring immune function; by measuring baseline levels (pre-challenge e.g. [15], [45]) or by measuring levels after an immune challenge is administered (induced e.g. [68]). In this study, we measured baseline levels. While it would have been informative to also investigate induced immunity, we had to balance the measurement of a diverse range of sexual signals across many individuals, against the logistical constraints of measuring both baseline and induced immunity. There is evidence that baseline and induced immune measures are correlated in *Teleogryllus*: pre-challenge haemocyte counts correlate with the degree of encapsulation of a nylon filament insert in both *T. oceanicus* and *T. commodus* [15], [25], [45]. The relationship between pre- and post challenge lysozyme activity in *T. commodus* is currently unknown. It is plausible that baseline levels reflect immunocompetence as a high baseline level of lysozyme could facilitate a more rapid immune response against invading pathogens. In scorpionflies, baseline lysozyme activity is positively correlated with the phagocytic capacity of the haemolymph (which aids in defence against bacteria and fungal spores) [70].

In summary, we found little evidence that females can assess a male’s immune function based on his calling. We did find a relationship between calling effort and lysozyme-like activity, however this must be interpreted with caution. More studies are needed before we can conduct a meta-analysis to test whether or not sexual traits in insects tend, on average, to signal male immune function. It is important that both significant and non-significant phenotypic correlations continue to be reported to avoid any publication bias in the scientific literature. Given that the fine-scale structures of the courtship and advertisement calls of *T. commodus* influence female choice, and that females presumably benefit from mate choice, male calls probably signal aspects of male quality other than the number of haemocytes or lysozyme activity. It is also likely that the different acoustic signals produced by males signal different information to females given that measures of advertisement and courtship call attractiveness were not related in our study.
